# Virus-Induced Cytoplasmic Aggregates and Inclusions are Critical Cellular Regulatory and Antiviral Factors

**DOI:** 10.3390/v12040399

**Published:** 2020-04-04

**Authors:** Oluwatayo Israel Olasunkanmi, Sijia Chen, James Mageto, Zhaohua Zhong

**Affiliations:** Department of Microbiology, Harbin Medical University, Harbin 150081, China; oolasunkanmi@aul.edu.ng (O.I.O.); lxj@hrbmu.edu.cn (S.C.); mageto.james@ku.ac.ke (J.M.)

**Keywords:** RNA granules, aggresome, autophagy, proteostasis

## Abstract

RNA granules, aggresomes, and autophagy are key players in the immune response to viral infections. They provide countermeasures that regulate translation and proteostasis in order to rewire cell signaling, prevent viral interference, and maintain cellular homeostasis. The formation of cellular aggregates and inclusions is one of the strategies to minimize viral infections and virus-induced cell damage and to promote cellular survival. However, viruses have developed several strategies to interfere with these cellular processes in order to achieve productive replication within the host cells. A review on how these mechanisms could function as modulators of cell signaling and antiviral factors will be instrumental in refining the current scientific knowledge and proposing means whereby cellular granules and aggregates could be induced or prevented to enhance the antiviral immune response in mammalian cells.

## 1. Introduction

Viruses lack their own metabolic machinery, therefore, to establish viral replicative complexes and achieve a robust and productive replication, they rely on essential cellular processes. Although some viruses can cause no apparent change in the infected cell, in most cases they can cause a wide range of structural, functional, and biochemical changes within the host cell [1]. As a countermeasure to these virus-induced cellular changes, host cells use structural, molecular, and or genetic mechanisms to control viral replication and spread by stimulating the formation of cellular inclusions like stress granules (SGs) and processing bodies (P-bodies). These cellular structures and their components serve as cytoprotective and survival factors that trigger intracellular RNA transcription and translation arrest or sequestrate vital cellular components required for viral replication. Alternatively, virus-infected cells can trigger membrane and cytoskeleton remodeling, which results in the formation of insoluble cytoplasmic aggregates, such as aggresomes, and autophagy [2]. Essential cellular and viral proteins required for effective viral replication, virus-induced stress proteins, and viral or cellular toxic proteins are sequestered and or degraded in these aggregates. Synergistically, aggresome and SG may be cleared by the cellular degradation machinery and autophagy to facilitate viral clearance and cellular recovery [2,3].

In light of recent knowledge, this review discusses the cytoprotective functions of cellular inclusions, aggregates, and their components, providing an overview on how these structures can function as an antiviral mechanism and a cellular signaling regulatory mechanism in virus-infected cells.

## 2. RNA Granules: Dynamic Modulators of Cellular Ribostasis and Antiviral Immunity

RNA granule is a structural term that broadly define a spectrum of entities involved in RNA transport and specific structures involved in RNA storage and/or decay machinery [4]. Some RNA granules are involved in normal cellular functions. However, as a cellular regulatory response or as a survival mechanism against several types of stimuli, the cells can trigger the formation of specific messenger RNA (mRNA) silencing *foci* [5,6].

The major types of RNA granules produced during viral infection are SGs and P-bodies [7]. Although similar, they differ in function and key components. The composition and assembly of both RNA granules has been reviewed [8]. SGs are in close apposition with P-Bodies. This possibly facilitates the rapid redistribution of a number of proteins, such as Argonaute protein 1 and 2 (AGO1 and 2), a catalytic component of RNA-induced silencing complex (RISC), and apolipoprotein B mRNA-editing enzyme, catalytic polypeptide-like 3G (APOBEC3G), involved in RNA silencing [9], from the P-bodies to SGs during cellular stress [10,11]. Functionally, P-bodies degrade transitionally repressed mRNAs, while SGs could either degrade or store mRNA for further use after cellular recovery [6]. Constitutively, P-bodies are irreversible silencing *foci* enriched with RNA degradation enzymes, decapping protein 1 and 2 (DCP) [12], 3′-5′ exosome and 5′-3′ exoribronuclease, and mRNA silencing mechanisms such as RNA interference (RNAi) and nonsense-mediated decay (NDM), while SGs are composed largely of translational initiation factors, preinitiation complex, and 40S ribosomal subunit. SGs and P-bodies share some components, such as eukaryotic initiation factor (eIF4E), tristeraprolin (TTP), RISC, APOBEC3, and poly(C)binding protein 2 (PCBP2) [4,13].

Virus infection is capable of eliciting stress within the host cells. In response to this, cells stimulate the assembly of RNA granules, SGs, and P-bodies, which modulate post-transcriptional RNA expression, cellular physiology, and homeostasis [14]. These RNA granules regulate cellular ribostasis and minimalize cellular expenditure of energy to promote cellular survival and prevent virus-induced stress damage [8,15]. Evidence of proteostasis activities of RNA granules, from proteomic analysis of mammalian SG core components, revealed that SGs sequestrate cellular translational factors and RNA binding proteins [16]. Presumably, this could reduce the availability of cytoplasmic components required for the translation of viral RNA transcripts, which can thwart viral replication. Alternatively, RNA granules could serve as cytoplasmic inclusion bodies that sequestrate a wide range of viral mRNA and protein components [17,18]. This may restrict viral replication depending on the type of protein and mRNA sequestrated, since components organized and silenced into condensed foci, P-bodies, and SGs can be degraded [2,14]. 

Furthermore, RNA granules contain host antiviral defense proteins. Cellular antiviral proteins, such as APOBEC3, (GTPase)-activating protein SH3-domain-binding protein 1 (G3BP1), retinoic acid-inducible gene 1 (RIG-1), and protein kinase R (PKR) are present within SGs and P-bodies [7,18]. They are involved in virus detection and silencing and in the activation of innate immunity [7,18]. It has been proposed that the interaction of APOBEC3 with cellular RNA-silencing pathways could possibly regulate cellular RNA functions at the post-transcriptional level [9].

Emerging evidence shows that RNA granules and innate immunity are connected at many levels. This interface may be principally due to the presence of G3BP1, RIG-1, and PKR in RNA granules, which serve as innate immunity pathogen sensors [7]. During infection, viral RNAs are detected by pathogen recognition receptors (PRR). This activates PKR that mediates the phosphorylation of eukaryotic initiation factor-α (eIF2α) on serine 51 to initiate the assembly of virus-induced SGs (vSG) [19]. These vSGs are capable of functioning as modulators of cell signaling by stimulating the production of cytokines [20].

RNA granules could serve as ‘sensing vehicles’ that link stress with interferon production [14]. Virus-induced SGs serve as antiviral vSG (avSG), a platform for efficient viral RNA detection. They selectively capture viral RNA transcripts and poly(A)-RNA to induce interferon (IFN) production [17,21]. Likewise, uncapped viral RNA derived from read-through transcription can be assembled into the avSG and sensed by RIG-1 to elicit an effective antiviral response within the host cells [22]. 

In addition, RNA granules could orchestrate other physiological responses that further enhance the antiviral state within the cell. This restricts viral replication by triggering cellular responses like autolysis and autophagy or switches the cell into a translationally silenced state to prevent and/or delay the translation of specific transcripts [14]. Analysis of endogenous mRNA localized in SGs by Zhang et al. (2011), using fluorescence microscopy recovery after photobleaching, showed that one-third of mRNAs localized within SGs are immobile, while other mRNAs can diffuse and reach equilibrium between SGs and cytosol [23]. Since SGs are enriched with RISC, co-localization of mRNA and RISC within the SGs functions as a cellular regulatory mechanism in the reprogramming of mRNA translation in the host cells [23]. 

Increasing evidence reveals the role of SGs and their components in deoxyribonucleic acid (DNA) virus infection. A Study by Rozelle et al. (2014) on the role of SGs in DNA virus-infected cells showed that SGs provide antiviral activity and restrict viral replication through by the formation of avSG. To determine if avSGs were formed during virus infection, human cells were infected with wild-type vaccinia virus (WT-VV) and stained with avSG- and G3BP1-specific antibodies. The results showed that avSG were formed in virus-infected cells and that these granules recruited host cellular proteins like G3BP1. Cells that formed avSG negatively regulated WT-VV gene expression. Further treatment of the virus-infected cells with β-thiosemicarbazone (IBT), an antiviral agent used for the treatment of smallpox, enhanced avSG formation. These results indicate the role of the formation avSG in the pharmacological inhibition of viral replication [24,25].

## 3. Aggresome: A Dynamic Modulator of Cellular Proteostasis and Antiviral Immunity

Aggresomes are perinuclear insoluble inclusion bodies that contain aggregated toxic misfolded/unfolded proteins sequestered for subsequent proteasome-machinery and/or autophagic degradation [24,26]. During virus infection, the synthesis of large amounts of viral protein causes accumulation of unfolded or misfolded proteins [27]. These toxic proteins are transported to the aggresomes for immobilization and subsequent degradation by the proteasome or autophagy machinery [24].

### 3.1. Mechanism of Aggresome Formation

**Figure 1:**
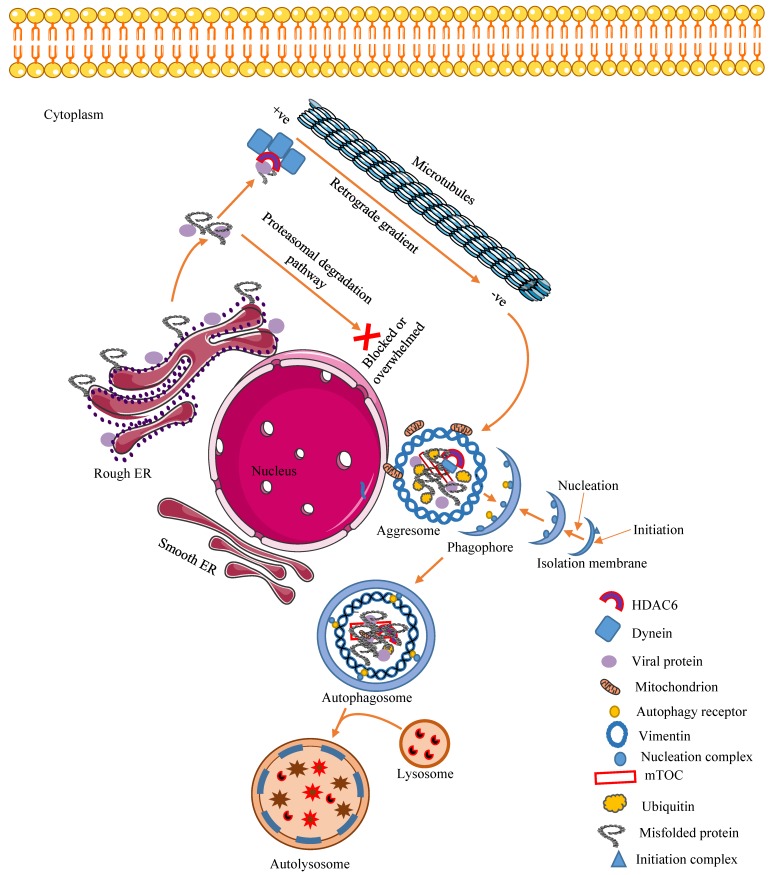
Virus-induced aggresome formation. The activation of the aggresome pathway is the result of an overwhelmed or blocked proteasomal degradation pathway. Aggresomes are formed around the MTOC at the perinuclear region of the cell by active minus-end-directed transport of proteins to the vimentin cage. HDAC6 is important for the aggregation of viral and or cellular misfolded/ubiquitinated proteins at the MTOC. These proteins are connected to dynein, a motor protein, and to HDAC6, a linker molecule with deacetylase activity. The aggresomes formed are cleared by an autophagosome–lysosome fusion event called aggrephagy. HDAC6, histone deacetylase 6, MTOC, microtubule organizing center, ER, endoplasmic reticulum. Figure adapted from [30]. Figure was drawn on smart.servier.com.

The formation of aggresome starts by the aggregation, in the cytosol, of unfolded or misfolded proteins produced in the endoplasmic reticulum (ER). These aggregates are recognized by histone deacetylase 6 (HDAC6), a unique enzyme that causes the removal of acetyl groups in histone and non-histone protein through its ubiquitin-binding domain and delivers them to the periplasmic region of the host cell by dynein-dependent retrograde transport on microtubules (MT). Olzmann and Chin identified parkin-mediated K63-linked polyubiquitination as the signal that couples misfolded proteins to dynein retrograde transport [28]. The retrograde transport of misfolded proteins along the MT involves the negative-end-directed dynein and HDAC6 complex. Once the proteins reach the perinuclear site, aggresomes are formed around the MT-organizing center (MTOC) and are surrounded by a vimentin cage [29]. The overall structure of the aggresomes depends on the sequestered substrate and the cell. Usually, most aggresomes appear as spherical (1–3 μm) or as extended ribbons [30] (Figure 1). 

Aggresomes are often mistaken for other protein aggregates. However, the essential feature that differentiates aggresomes from other types of protein aggregates is their MT dependency and co-localization with centrosomes at the perinuclear region of the cell. 

Although the central process of aggresome formation is the acetylation of HDAC6 [31], a number of other factors have been associated with the formation of aggresomes. These include p62/sequestosome 1 (SQSTM1), an autophagy cargo receptor responsible for the type of protein degradation via aggresomes or autophagy degradation pathways [32], protein linking integrin-associated protein (IAP) to the cytoskeleton (PLIC), a ubiquitin-like protein that binds the ubiquitin interacting motif (UIM) of proteasomal subunit S5, ataxin 3, and deubiquitinating enzymes [33,34]. Also, some cellular organelles and proteins, such as mitochondria, heat-shock proteins (HSP), and Golgi bodies are recruited to the site of aggresome formation.

### 3.2. Cellular Regulatory and Antiviral Effects of Aggresomes and Their Components

Aggresomes were first described as assembly sites for misfolded cystic fibrosis transmembrane conductance receptors [35]. However, it is now believed that aggresome formation is a general cellular regulatory response to toxic proteins. The raising question is whether agressomes are part of the cellular regulatory and antiviral response in virus-infected cells. A study has shown that HDAC6-mediated aggresomes target and localize intracellular invasive viruses and/or viral proteins for clearance by autophagic degradation [36]. Similarly, HDAC6, an essential component of the aggresomal pathway, upon viral entry, modulates the intracellular trafficking of cytoplasmic contents and the local reorganization of MT. Ultimately, this restricts the spread of viruses and prevents their pathogenesis [37]. Experiments carried out by Husain et al. (2014) demonstrated that the co-localization of Influenza A virus (IAV) ribronucleoprotein (RNP) complex with acetylated HDAC6 and MT exerts an antiviral effect that inhibits the trafficking of viral replicative proteins to the assembly site, which limits the release of viral progeny [38]. Interestingly, the inhibition of the acetylation of HDAC6 and MT by tubacin increased the assembly and release of IAV progeny in a dose-dependent manner [38]. Aggresome formation is a cytoprotective cellular response. There is a close correlation between aggresome formation and cell survival [39]. More importantly, aggresomes concentrate cellular chaperone/HSP, elements of proteasomal degradation pathways, in order to degrade aggregated viral and cellular proteins [40]. Since viruses and viral proteins appear as foreign to the host and may appear as misfolded proteins, there is the possibility that when recruited to the aggresomes, they undergo degradation [2]. Likewise, during viral assembly, the sub-viral nucleoprotein complex needs to travel to the plasma membrane for assembly. These proteins are similar to aggregates that are transported to aggresomes by dynein motors [2,41]. Arguably, since aggresomes are not just static garbage or depository bodies, they recruit cytoplasmic refolding and degradation protein, such as ubiquitination enzymes, proteasome components, Hsp70, and Hsp27, to aid the clearance of these aggregated proteins [42]. The aggregated proteins can also be cleared through HDAC6-mediated actinomyosin- and autophagy-dependent aggresome degradation [43].

An important regulator of the aggresomal pathway is HDAC6, a member of class IIb histone deacetylases responsible for the homeostasis of the cellular MT apparatus [44]. Cells deficient in HDAC6 cannot form aggresomes [45,46]. HDAC6 is a key enzyme for clearing unubiquitinated proteins that fail to enter the proteasome pathway [47,48]. Of note is an HIV accessory protein, viral infectivity factor (vif), which is post-translationally mono-unubiquitinated and it is not recruited to the proteasome degradative pathway [49,50]. However, HDAC6 interacts with vif or APOBEC3 and interferes with vif–APOBEC3 interaction through its binder of ubiquitin zinc finger (BUZ) domain, impairing the incorporation of vif into the nascent virion [48]. Trans-activator of transcription (Tat) protein, an essential protein for HIV-1 transcription and replication, has been shown to be regulated by HDAC6 [51]. HDAC1 binds and interacts with Tat at Lys^28^ in an MT-dependent manner. Inhibition of HDAC1 in HIV-1-infected cells has been shown to increase Tat transactivation activity, which invariably increased HIV transcription and replication [51]. Also, HDAC6 modulates the cellular endocytic uptake during virus infection. For instance, HDAC6 activity has been reported to prevent HIV-1 envelope-dependent cell fusion and infection [48]. It modulates the selective up-regulation of type 1 IFN production [52] and promotes viral RNA sensing by activating RIG-1, MAVIS-, Interferon Regulatory Factor 3 (IRF-3), and Nuclear Factor kappa B (NF-κB) signaling pathways [53,54].

The sequestration of viral proteins into inactive aggregates reduces their activity. Findings by Kajitani et al. (2013), showed that human papillomavirus 18 (HPV 18)-infected cells formed aggresome-like compartments at the perinuclear site of the host cells. The major viral oncoproteins E6 and E7 were recruited and sequestrated as an insoluble fraction in these aggresome-like compartments. These compartments, like classical aggresomes, associate with the vimentin cage and are assembled by dynein-dependent retrograde transport along microtubules filaments [55]. The sequestration of viral proteins renders them susceptible to autophagy and results in suppressed viral pathogenesis.

## 4. Autophagy: A Dynamic Modulator of Cellular Functions and Antiviral Immunity

Autophagy is a cell survival, defense mechanism by which the cell delivers sequestered cytoplasmic organelles, damaged proteins, or pathogens for lysosomal degradation in a compartmentalized double-membrane vesicle called autophagosome [56]. Autophagosome, a key structure in autophagy, serves as a cargo that delivers cytoplasmic substrates to lysosomes for degradation [57]. Autophagy provides a quality control system for the removal of damaged organelles and misfolded, long-lived, or toxic protein aggregates from the cytoplasm [58,59]. It aims to substantially promote cellular survival in response to endogenous and exogenous stimulations such as starvation [59,60] and viral infection [61]. Autophagy is a lysosome-dependent mechanism mediated by the formation of an isolation membrane, the phagophore [62]. Autophagosomes arise from phagophores, which elongate to engulf autophagic substrates in bulk or selective processes [60]. The fusion of autophagosomes with lysosomes or endosomes forms autolysosomes, within which degradation occurs [63].

Autophagy is active at a basal level to regulate cellular homeostasis [64]. It is a highly regulated process, suppressed by target of rapamycin (TOR) kinase [24]. However, during starvation, stress, or pathological conditions, it can be substantially activated to promote cellular survival [65], support energy demands, or prevent microbial pathogenesis [59].

### Autophagy Regulates Cellular and Antiviral Responses to Virus Infection

During viral infection, autophagy serves as a catabolic process that controls infection at several levels. It captures and destroys viruses or viral components, a process called virophagy [66,67]. Autophagy promotes antigen presentation or activates adaptive immune signaling by delivering viral nucleic acid to toll-like receptors (TLR) and major histocompatibility complex (MHC) class I and II molecules. For example, a study showed that recognition of vesicular stomatitis virus (VSV) [68] and HIV [69] by TLR7 in plasmacytoid dendritic cells requires the transport of cytosolic viral replicative intermediates into lysosomes via autophagy. Autophagy has been found to be involved in the presentation of viral antigens by MHC class I molecules in herpes simplex virus (HSV)-, HIV-1-, and Epstein-Bar virus (EBV)-infected dendritic or lymphoblast cells [70,71,72]. Likewise, autophagy regulates innate immunity by controlling mitochondria quantity and the production of reactive oxygen species (ROS) during microbial infection [56,73]. The process by which the cell directs autophagy as an innate component of immunity against viruses, is known as xenophagy [74]. It is an efficient antiviral response against positive- and negative-stranded RNA viruses and DNA viruses [75]. 

Cells trigger autophagy at the different stages of viral replication, that is, the early-phase, i.e., during virus attachment and entry, mid-phase, and late-phase of infection [75]. For instance, at the early stage of viral infection, the cell responds to HIV-1 infection by activating the autophagic response through the interaction between the C-terminal domain on the fusogenic gp41 subunit of the HIV-1 viral envelope protein and cluster of differentiation 4^+^ (CD4^+^) and C-X-C chemokine receptor 4 (CXCR4) in T-cells [76]. Similarly, autophagy can be induced at the mid-stage of virus infection by direct interaction of viral structural or non-structural proteins with cellular organelles or proteins. Autophagy can be induced as a cellular response to endogenous stress, such as ER stress or oxidative stress, in virus-infected cells [77]. A typical example is hepatitis C virus (HCV)-induced ER stress in hepatoma cells, which results in the accumulation of unfolded proteins. As a cellular countermeasure to improve cellular survival and to inhibit viral replication, the cells mounts a substantial unfolded protein response (UPR) that activates a robust autophagic process [78,79].

Autophagy-related protein (ATG), one of the key components of autophagy induction, functions as a regulator of innate immunity and other cellular signaling pathways, even in the absence of autophagosomes or autolysosomes, during viral replication. For example, ATG5, an autophagy key protein involved in the elongation of the phagophoric membrane in autophagic vesicles, conjugates with ATG12 to form the ATG5–ATG-12 conjugate. This conjugate is capable of regulating IFN type 1 signaling pathways mediated by RIG-1 and IFN-β promoters through their interaction with caspase recruitment domains (CARDs).

## 5. Interplay between Virus-Induced Aggregates and Inclusions

Mammalian cells face different types of stress including virus-induced stress. In dealing with this, eukaryotic cells activate several mechanisms to regulate the effect of this stress, inhibit the stressor, and promote cell survival [80]. Intracellular aggregates and inclusions serve as stress suppressors and cellular quality control strategies to maintain cellular integrity and viability. These cellular structures share some characteristics, and their mechanism of action and activation are connected at several levels.

Infection with a wide range of DNA and RNA viruses has been reported to activate the autophagic response, as inferred from the increased number of autophagic vesicles in virus-infected cells. By cytoplasmic organelles that gather cellular contents into double-membrane vesicles, the autoghagosomes, autophagy specifically delivers aggresomes and SGs for degradation via autolysosomes. Considering the dynamic nature of autophagy, it is important to gain insight on how SGs and aggresomes coordinately communicate with autophagy to prevent viral pathogenesis and how they are subsequently cleared by it. Both mechanisms will be discussed in the following paragraphs.

Various mechanisms have been reported that activate the autophagic response in virus-infected cells. Virus-induced autophagy can be triggered, for instance, when a virus binds to a receptor on the host cell. An example is CD46 surface receptor for measles virus and adenovirus [62,81]. Besides attachment, HCV, coxsackievirus B (CVB), and some nucleocytoplasmic DNA viruses—hepatitis B virus (HBV) and varicella zoster virus (VZV)—induce autophagy inside the host cells by activating stress responses like ROS or ER stress [77]. Stress to the ER disrupts the global protein production and folding. Misfolded proteins are aggresome-prone. To restore ER protein homeostasis, misfolded or unfolded proteins are retrotranslocated from the ER to the cytosol [82] for subsequent degradation by proteasomes and autophagy [83].

**Figure 2:**
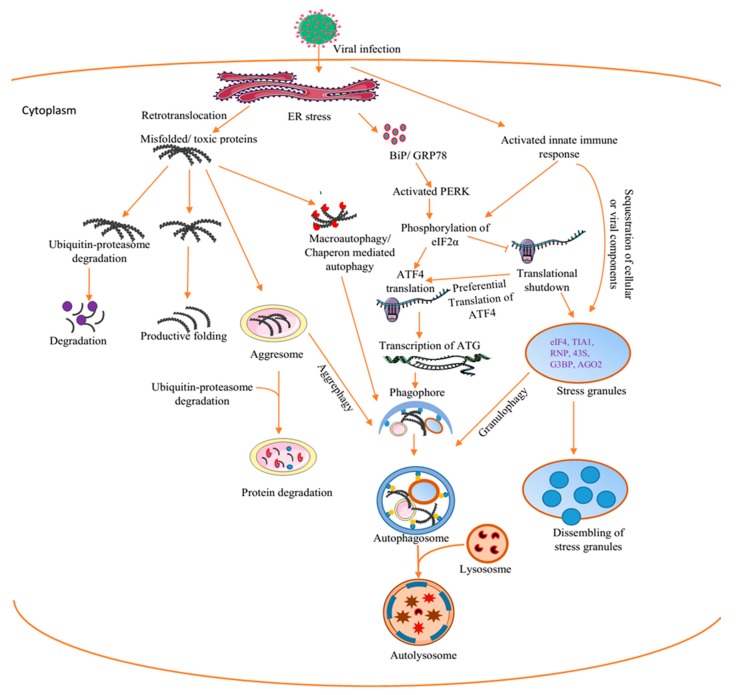
Interplay between virus-induced cellular aggregates and inclusions. The replication of viruses triggers ER stress. This causes the release of BiP/GRP79 into the cytoplasm and the subsequent activation of the unfolded protein response (UPR). This could ultimately lead to the clearance of toxic proteins by autophagy. Alternatively, virus infection directly activates innate immune responses capable of causing translational shutdown and stress granules (SGs) formation. Cellular and or viral proteins could appear as misfolded or toxic proteins and are recruited to the chaperon pathway for productive refolding and/or to the proteasomal pathway. However, when these pathways are blocked, the misfolded proteins are sequestrated in the aggresomes and are cleared by autophagy–aggrephagy. BiP/GRP78, binding immunoglobulin protein/glucose-regulated protein 78, ATF4, activating transcription factor 4, eIF2α, eukaryotic initiation factor 2 alpha, ATG, autophagy-related protein, RNP, ribronucleoprotein, AGO2aArgonaute, G3BP-1, Ras GTPase-activating protein-binding protein-1, PERK, protein kinase-like endoplasmic reticulum kinase. Figure was drawn on smart.servier.com.

Likewise, to avert ER stress-induced cell death, the cells activate UPR, PKR-like ER (PKR), activating transcription factor 6 (ATF6), and inositol-requiring protein 1α (IRE1α) [84]. Activated PERK mediates the phosphorylation of eIF2α. Phosphorylated eIF2α supresses cellular translation and prevents the formation of eIF2–guanosine triphosphate–initiator methionyl-transferRNA (eIF2–GTP–met-tRNAi) [85,86]. Classically, the formation of SGs is triggered at this step. Although the phosphorylation of eIF2α causes a temporal global translational shutdown, there is a preferential translation of activation transcription factor 4 (ATF4) [87]. The eIF2α/ATF4 signaling pathway fine-tunes the upregulation of ATG, essential for stress-induced autophagy [87]. The activation of PERK connects SG to autophagy [87,88] (Figure 2).

Since SGs and aggresomes are space-filling entities, if they exceed a certain threshold, they could physically interrupt normal cellular functions. Hence, to prevent this, aggresomes and SGs components dissassemble in multiple steps that involve the production of smaller fragments that are cleared by chaperon-dependent degradation or autophagy [16]. Alternatively, SGs and aggresomes can be targeted, via autophagy receptors [89], for selective autophagic clearance, termed granulophagy and aggrephagy, respectively [90]. Autophagy receptors like SQTM1/p62 and calcium-binding, coiled-coil domain-containing protein 2/nuclear dot 10 protein 52 (CALCOCO2/NDP52) have been reported to mediate selective autophagy during viral pathogenesis [91]. For instance, in Coxsackievirus A (CVA)-infected cells, granulophagy is mediated by the interaction between the ubiquitin-associated (UBA) domain of p62 and the ubiquitin-binding domain (UBD) of HDAC6, a component of viral RNA-induced SGs [92]. Selective autophagy double-membrane vesicles, the autophagosomes, encapsulate and deliver their cargo to lysosomes for degradation.

Several factors appear to specifically target SGs for autophagic degradation. Aside from canonical ATG, granulophagy requires valosin-containing protein, an ATPase that degrades SG components in autophagy [16,90]. Other factors are the return of the sequestrated mRNA to active translation and the decapping of SG mRNA [16,93]. 

Some viruses and viral proteins share structural motifs that are similar to amino acid cargo recognized by dynein; this suggests that they are aggresome-prone and susceptible to degradation by autophagy. A recent study by Mohamud et al. (2019) showed that the autophagy receptors CALCOCO2 and p62 regulate CVB3 pathogenesis through the interaction with CVB3 viral protein 1 (VP1) that undergoes ubiquitination during infection. Further investigation revealed that both receptors appear to have a role in virophagy through interaction with VP1. Knockdown of p62 resulted in elevated viral titers [94]. Specifically, ubiquitylated proteins may have a role in targeting the selective autophagy of aggresomes and SGs [95].

## 6. Virus Exploitation of Cellular Inclusions and Aggregates

A striking observation is that the formation of aggresomes and SGs and autophagy not only inhibit viral pathogenesis but also are employed by viruses to subvert host proteins involved in antiviral signaling. Viruses employ different mechanisms to manipulate and co-opt these cellular structures and their components for effective replication. For instance, active viral replication is not commonly associated with the presence of SGs [2]. Therefore, in order to survive, viruses have to develop mechanisms to evade or prevent the formation of RNA granules. Viral evasion mechanisms include prevention of the assembling of RNA granules and dissolution of existing ones [16]. Alternatively, viruses can subvert SG antiviral proteins. This can compromise granular integrity and antiviral efficiency. For example HSV, dengue virus, and HIV-1 viral proteins block SG formation by binding to the SG core proteins T-cell intracellular antigen (TAI-1) and G3BP1 [96,97]. Likewise, HIV-1 vif interacts with APOBEC3, a component of SGs, to cause its proteasomal degradation. This viral protein subverts the antiviral activity of APOBEC3 in HIV-infected cells to promote viral pathogenesis [48]. Equally, RISC-mediated antiviral activity of P-bodies is strongly inhibited by HIV-1 vif, which is capable of disrupting P-body structural integrity by allowing the virus to replicate virtually undisturbed [98].

Other viruses can cause the dispersion of existing RNA granules structural components [14]. An experimental report by Dougherty et al. (2015) demonstrated that poliovirus induces SG formation during the early phase of infection while at mid phase, it inhibits SG formation and disperses P-body components. Inhibition of SG formation during poliovirus infection has been linked to cleavage of G3BP1 in SGs by the viral protein 3C^pro^ [99]. 

Evidence suggests that the assembly of several cytoplasmic viruses in mammalian cells occurs at an intracellular site called the ‘virus factory’ or ‘viroplasm’ [2]. The virus factory contains cellular and viral proteins required for viral genomic replication and morphogenesis of new virions. In some instances, the viroplasm has been compared to the cellular aggresome. The replication and assembly of poxvirus have been demonstrated to take place in a virus factory that resembles the aggresome [2,24,100]. Some virus factories contain similar components found in aggresomes, such as chaperon/ heat shock protein, proteases, and MTOC [2]. An MTOC-dependent virus factory has been observed in togavirus-, flavivirus-, and buyanvirus-infected cells [2]. Virus factories are sometimes functionally comparable to cellular aggresomes. This highlight the possibility that aggresomes can be used as virus replicative and assembly sites [2,24].

Experimental reports suggested that some viruses can induce the accumulation of cellular antiviral proteins and subsequently facilitate their degradation by selective autophagy (aggrephagy). Murid cytomegalovirus (MCMV)—a herpesvirus—M45 protein induces the sequestration of two cellular signaling proteins, NF-κB essential modulator (NEMO) and receptor-interacting protein kinase 1 (RIPK1), through its ‘induced protein aggregation motif’ (a conserved protein motif whose homologous is present in several human herpesviruses) and subsequently facilitates their degradation by autophagy to evade the host immune response in infected cells [101].

Autophagy, a cellular process aimed to clear pathogens [102], can be subverted during virus replication. Mechanisms of viral evasion of autophagy include exploitation of secretory autophagy to exit the cells, non-lytic shedding, and blockage of the autophagic flux. For instance, a report by Granato et al. (2014) showed that EBV, a gammaherpes virus associated with non-Hodgkin’s B cell lymphoma, blocks the autophagic flux at the final step during reactivation from latency [102]. A similar report by Kembal et al. (2010) showed an increase in the number of large autophagy-like double-membraned vesicles, megaphagosomes, and the accumulation of the autophagy receptor p63 in CVB3-infected cells. This suggests that CVB3 blocks a later stage in autophagy formation [103]. The double-membraned vesicles megaphagosomes serve as a scaffolding for viral RNA replication and immune escape [104,105]. Likewise, viruses can subvert autophagic responses by targeting one of the autophagy proteins. This phenomenon has been reported in Kaposi’s sarcoma-associated herpesvirus (KSHV)-infected cells. KSHV is a human herpes virus associated with multiple cancers, whose oncogenic protein v-cyclin interacts with ATG3 to subvert autophagic responses, blocks senescence, and enhances viral replication [106]. However, unlike other viruses that block autophagy, some viruses activate autophagy to benefit from autophagy-dependent processes. For instance, Dengue virus (DENV), a mosquito-borne single-stranded RNA virus that causes haemorrhagic fever, benefits from autophagy-specific processes like lipophagy, a form of autophagy that serves as an alternative to lipid metabolism [104]. DENV co-localizes with lipid droplets within the autolysosome, which correlates with an increase in DENV replication [107].

## 7. Conclusions and Future Perspectives

This review shed light on aggresomes, SGs, and autophagy as cellular regulatory structures and antiviral mechanisms. It highlights that these virus-induced aggregates and their components could play a dual role as elements of the antiviral innate immune response and as regulators of other cellular activities. They act by sequestrating and/or degrading cellular or viral replicative components to maintain a homeostatic and antiviral state within cells. The sequestrated substances become trapped and are sorted for degradation or become unavailable for the generation of new virus particles. Alternatively, cellular aggregates, like SGs and aggresomes, serve as protective structures and storage sites, where important active cellular components and structures are sequestrated in order to prevent their rapid degradation during virus infection.

Despite our current, developing knowledge of the mechanisms and functions of virus-induced cellular aggregates and inclusions, mechanisms of interactions between these aggregates/inclusions and viruses are still to be deciphered to obtain a complete view of host–virus interactions at the cellular level. Furthermore, since accumulating evidence suggests that these structures can be subverted to enhance viral replication or can be used as viral replicative platforms, the recognition of key cellular and viral regulatory proteins that promote viral subversion of these aggregates and inclusions will provide a significant advancement for the development of new antiviral therapeutic strategies and approaches to fight viral infections.
